# Diet Quality and Risk of Lung Cancer in the Multiethnic Cohort Study

**DOI:** 10.3390/nu13051614

**Published:** 2021-05-12

**Authors:** Song-Yi Park, Carol J. Boushey, Yurii B. Shvetsov, Michael D. Wirth, Nitin Shivappa, James R. Hébert, Christopher A. Haiman, Lynne R. Wilkens, Loïc Le Marchand

**Affiliations:** 1 Cancer Epidemiology Program, University of Hawaii Cancer Center, Honolulu, HI 96813, USA; cjboushey@cc.hawaii.edu (C.J.B.); yshvetso@cc.hawaii.edu (Y.B.S.); lynne@cc.hawaii.edu (L.R.W.); loic@cc.hawaii.edu (L.L.M.); 2 College of Nursing, University of South Carolina, Columbia, SC 29208, USA; wirthm@email.sc.edu; 3 Department of Epidemiology and Biostatistics, Arnold School of Public Health, University of South Carolina, Columbia, SC 29208, USA; shivappa@email.sc.edu (N.S.); jhebert@mailbox.sc.edu (J.R.H.); 4 Department of Nutrition, Connecting Health Innovations LLC, Columbia, SC 29201, USA; 5 Keck School of Medicine and Norris Comprehensive Cancer Center, University of Southern California, Los Angeles, CA 90033, USA; christopher.haiman@med.usc.edu

**Keywords:** cohort, diet quality, lung cancer, multiethnic population

## Abstract

Diet quality, assessed by the Healthy Eating Index-2015 (HEI-2015), the Alternative Healthy Eating Index-2010 (AHEI-2010), the alternate Mediterranean Diet (aMED) score, the Dietary Approaches to Stop Hypertension (DASH) score, and the Dietary Inflammatory Index (DII^®^), was examined in relation to risk of lung cancer in the Multiethnic Cohort Study. The analysis included 179,318 African Americans, Native Hawaiians, Japanese Americans, Latinos, and Whites aged 45–75 years, with 5350 incident lung cancer cases during an average follow-up of 17.5 ± 5.4 years. In multivariable Cox models comprehensively adjusted for cigarette smoking, the hazard ratios (95% confidence intervals) for the highest vs. lowest quality group based on quintiles were as follows: 0.85 (0.77–0.93) for HEI-2015; 0.84 (0.77–0.92) for AHEI-2010; 0.83 (0.76–0.91) for aMED; 0.83 (0.73–0.91) for DASH; and 0.90 (0.82–0.99) for DII. In histological cell type-specific analyses, the inverse association was stronger for squamous cell carcinoma than for adeno-, small cell, and large cell carcinomas for all indexes. There was no indication of differences in associations by sex, race/ethnicity, and smoking status. These findings support that high-quality diets are associated with lower risk of lung cancer, especially squamous cell carcinomas, in a multiethnic population.

## 1. Introduction

High-quality diets, assessed by pre-defined indexes, may lower risk for various health outcomes in epidemiological studies [1,2,3]. Several quality indexes, including those based on dietary guidelines or recommendations and the inflammatory potential of diet, have been applied and found inversely associated with risk of lung cancer [4,5], which is the leading cause of cancer deaths in the US [6]. Since cigarette smoking is a strong risk factor, is related to overall diet quality [7,8,9], and is a strong pro-inflammatory agent [10], most studies of diet quality and lung cancer comprehensively controlled for smoking and stratified the association by smoking status. However, potential differences across racial/ethnic groups and histologic subtypes of lung cancer have rarely been examined.

In the Multiethnic Cohort (MEC), the overall quality of participants’ diets has been assessed using five indexes, the Healthy Eating Index-2015 (HEI-2015), the Alternative Healthy Eating Index-2010 (AHEI-2010), the alternate Mediterranean Diet (aMED) score, the Dietary Approaches to Stop Hypertension (DASH) score, and the Dietary Inflammatory Index (DII^®^) [11,12]. In the current study, we investigated the associations between the five diet quality indexes and incident primary invasive lung cancer in the MEC and whether the associations varied by sex, race/ethnicity, and histologic subtype.

## 2. Materials and Methods

### 2.1. Study Population

The MEC is a population-based, prospective cohort founded to study the relation of lifestyle, especially diet, and genetic factors to cancer and other chronic diseases [13]. Between 1993 and 1996, more than 215,000 adults (55% women) aged 45 to 75 years entered the cohort by returning a self-administered, 26-page mailed questionnaire that included a detailed dietary assessment and consent to participate in the study. Study participants were mostly African Americans (16%), Native Hawaiians (7%), Japanese Americans (26%), Latinos (22%), and Whites (23%) living in Hawaii or Southern California, by design through targeted recruitment. The institutional review boards at the University of Hawaii and the University of Southern California approved the study protocol (approval no. CHS9575 and HS-17-00714, respectively). For the current analyses, we excluded participants who did not self-report one of the five racial/ethnic groups (*n* = 13,987), had lung cancer prior to cohort entry reported on the baseline questionnaire (*n* = 438) or from tumor registries (*n* = 287), or reported extreme diets based on total energy intake or its components (*n* = 8210). Specifically, we computed a robust standard deviation (RSD) of total energy intake based on the truncated normal distribution after excluding the top and bottom 10% tails. Then, we excluded all individuals outside the ranges of mean ± 3 RSD. We excluded individuals with extreme fat, protein, or carbohydrate intakes using a similar procedure [14]. We further excluded participants with missing information on smoking (*n* = 7509) and other important covariates, including education, body mass index, and physical activity (*n* = 5893). As a result, a total of 179,318 participants were included in the current analysis.

### 2.2. Dietary Assessment and Covariates

At cohort entry, dietary intake during the previous year was assessed by a quantitative food frequency questionnaire (QFFQ) containing over 180 food items. The QFFQ was developed from 3-day measured food records collected from 60 men and women of each ethnic group [13]. A calibration study demonstrated satisfactory correlations for nutrients as energy densities (0.57–0.74) between the QFFQ and three 24-h recalls for each sex–racial/ethnic groups [15]. On the baseline questionnaire, participants also reported socio-demographic and health- and lifestyle-related information including race/ethnicity, smoking history, medical history, physical activity, and body weight and height.

### 2.3. Diet Quality Indexes

The Dietary Patterns Methods Project (DPMP) computed four dietary indexes for all MEC participants using a standardized protocol, which was developed for and applied to three large cohorts, the MEC, the NIH-AARP Diet and Health Study, and the Women’s Health Initiative Observational Study (WHI-OS) [11]. In brief, the HEI-2015 quantified adherence to the 2015–2020 Dietary Guidelines for Americans (13 components, 0–100 points) [16]. The HEI-2015 replaced the 2010 version of the HEI initially computed in the DPMP [17]. The AHEI-2010 identified dietary patterns consistently related to lower risk of chronic disease (11 components, 0–110 points) [18]. The aMED was an adaptation for use in a US population of the Mediterranean Diet score (MDS), which was associated with lower mortality in Mediterranean populations (nine components, 0–9 points) [19]. The DASH captured the diet in two DASH feeding trials that tested the role of dietary patterns on blood pressure (eight components, 8–40 points) [20]. In addition to these four indexes, the DII was calculated to assess the overall inflammatory potential of an individual’s diet [12]. On the basis of published evidence, a total of 45 dietary parameters correlated with blood inflammatory markers were scored to determine an inflammatory effect score [21]. For the MEC, 28 of the 45 dietary components were available for inclusion in the DII calculation [12]. While higher scores of the four indexes reflect better diet quality, higher DII shows more pro-inflammatory diet and thus reflects poorer quality of diet.

### 2.4. Case Ascertainment

Lung cancer cases were identified by linkage of the cohort to the Surveillance, Epidemiology, and End Results Program (SEER) tumor registries in Hawaii and California. Deaths were ascertained by linkage to death files in both states and the National Death Index. Case and death ascertainment were completed through December 31, 2014. Lung cancers were defined based on the International Classification of Diseases for Oncology (ICD-O-3) and ICD-10, C34 [22]. Major histological cell types of lung cancer were categorized using morphology codes reported by Lewis et al. [23]. During an average follow-up period of 17.5 years, 5350 lung cancer cases were identified among eligible participants.

### 2.5. Statistical Analysis

Cox proportional hazards models with age as the time metric were used to calculate hazard ratios (HRs) and 95% confidence intervals (95% CIs) of lung cancer according to the dietary scores in men and women separately and combined. Dietary scores were divided into quintiles based on their distributions across the entire cohort. The lowest quintile of the score (poorest diet quality) was set as a reference group for the HEI-2015, the AHEI-2015, the aMED, the DASH, while the highest quintile of the DII (most pro-inflammatory diet) served as a reference group. Trend variables for the indexes were assigned the sex- and race/ethnicity-specific medians for quintiles. We used a comprehensive smoking model developed to investigate tobacco use and lung cancer incidence in the MEC [24]. The model explicitly contained smoking status (never, former, current), average number of cigarettes per day, squared average number of cigarettes per day, number of years smoked (as a time-dependent variable), number of years since quitting (as a time-dependent variable), and interactions of race/ethnicity with smoking status, average number of cigarettes per day, squared average number of cigarettes per day, and number of years smoked. The model was further adjusted for age at cohort entry, family history of lung cancer (yes, no), physical activity (hours of moderate or vigorous activity per day), and total energy intake (log-transformed kcal/day) as covariates and race/ethnicity, education (≤12th grade, vocational school/come college, ≥college graduate) and body mass index (BMI, ≤25, 25−29.9, ≥30 kg/m^2^) as strata variables for the purpose of computing baseline hazard functions. For the HEI-2015 and DASH models, alcohol consumption (g/day) was additionally adjusted for as a covariate. The proportional hazards assumption was verified by Schoenfeld residuals [25]. Since the association was similar in men and women, we combined men and women for subgroup analysis, with adjustment for sex as a strata variable.

We fit the models stratified by race/ethnicity and smoking status and for each histologic cell type separately. Tests for heterogeneity between subgroups were tested by the Wald statistics for cross-product terms of trend variables and subgroup indicator. Tests for heterogeneity by histological cell type were based on a Wald statistics comparing associations between cell type using competing risk methodology and an augmented data approach [26,27]. In supplemental analyses, dietary indexes were updated as time-dependent variables using data from the MEC 10-year follow-up survey (2003–2008) that was available for 82,119 (46%) of the 179,318 participants. All analyses were performed by using SAS 9.4 software (SAS Institute, Inc., Cary, NC, USA).

## 3. Results

The five dietary indexes were significantly correlated with each other (all Ps < 0.001); the absolute values of all Pearson correlation coefficients were between 0.48 and 0.77 (Table 1). The correlations were similar between men (0.51–0.77) and women (0.48–0.76) and across racial/ethnic groups (0.42–0.78 in African Americans, 0.48–0.83 in Native Hawaiians, 0.54–0.79 in Japanese Americans, 0.44–0.73 in Latinos, and 0.47–0.78 in Whites).

Compared to those in the lowest quintile of HEI-2015, men and women in the highest quintile were more likely to be older, more educated, never smokers, less obese, and more physically active (Table 2). Similar trends were found with the other four dietary indexes (Supplementary Table S1). Men and women with the highest HEI-2015 score tended to be African American and White (Table 2), while those in the highest AHEI-2010 and aMED groups were more likely to be Japanese American, and those with the most beneficial DASH and DII scores tended to be White (Supplementary Table S1).

Among both sexes, all dietary indexes were associated with lower risk of lung cancer, except for the DASH and DII in men, for which only the fourth and third quintiles showed a significant reduction in risk, respectively (Table 3). Overall, the risk reductions were greater in women (10–27%) than in men (8–13%), comparing the highest vs. lowest groups of diet quality. However, tests for heterogeneity did not indicate sex differences in the associations (all Ps for heterogeneity ≥ 0.23). In men and women combined, the inverse association was statistically significant for all five indexes, with a range of 10 to 17% decrease in risk across extreme quintiles.

Of the total lung cancer cases, 40% were adenocarcinoma, 20% were squamous cell carcinoma, 10% were small cell carcinoma, and 3% were large cell carcinoma. Across the indexes, the inverse associations were stronger for squamous cell carcinoma (19–29% decrease in risk) and other/unspecified cell type (24–27% decrease in risk) than for adeno-, small cell, and large cell carcinomas (Table 4, all Ps for heterogeneity < 0.001). For the latter three subtypes, a significant association was found only between aMED and small cell carcinoma.

In racial/ethnic-specific analyses, all five indexes were inversely associated with lung cancer risk in Whites, while only some of the indexes were significantly associated in the other racial/ethnic groups (Supplementary Table S2). Nevertheless, tests for heterogeneity across the five racial/ethnic groups were not statistically significant (Ps for heterogeneity ≥ 0.29). The associations did not vary across never, former, and current smoking status at cohort entry (Ps for heterogeneity ≥ 0.19, Supplementary Table S3). When updating diet quality indexes with data from the 10-year follow-up questionnaire, the associations remained similar (Supplementary Table S4). The risk reduction ranged 11 to 20% in all participants and, among the respondents to the 10-year questionnaire, 7 to 25% for the incident cases occurring afterward.

## 4. Discussion

In this large multiethnic population, high-quality diets assessed using five indexes were associated with lower risk of lung cancer in both men and women, although the trend tests for the DASH and DII did not quite reach statistical significance in men with adjustment for smoking and potential confounders. In men and women combined, the inverse association was stronger for squamous cell carcinoma than for adeno-, small cell, and large cell carcinomas. There was no evidence for heterogeneity in the associations by race/ethnicity and smoking status.

Previous observational studies found an inverse association between diet quality and lung cancer risk. A meta-analysis of four case-control and four cohort studies reported an overall 19% decreased risk (95% CI: 0.75–0.86) comparing the highest vs. lowest categories of healthy dietary patterns, which were defined by a data-driven approach or an index-based approach [4]. The corresponding risk reduction was 11% (95% CI: 0.63–1.27) for non-smokers, 26% (95% CI: 0.62–0.89) for former smokers, and 14% (95% CI: 0.79–0.93) for current smokers [4]. The meta-analysis included a large US cohort, the NIH-AARP Diet and Health Study that participated in the DPMP. In this cohort, the HR (95% CI) for the highest vs. lowest quintiles was 0.83 (0.77–0.89) for HEI-2010, 0.86 (0.80–0.92) for AHEI-2010, 0.85 (0.79–0.91) for aMED, and 0.84 (0.78–0.90) for DASH [28], which was comparable with the current results in the MEC. For histologic cell types of lung cancer in the NIH-AARP Diet and Health Study, the inverse association was observed for adenocarcinomas and squamous cell carcinomas but not for small cell carcinomas [28]. In another DPMP cohort of women only, the WHI-OS, the four indexes were not associated with lung cancer incidence overall but showed a protective association against squamous cell carcinoma, with an HR (95% CI) of 0.56 (0.33–0.96) for HEI-2010, 0.42 (0.24–0.76) for AHEI-2010, 0.65 (0.39–1.08) for aMED, and 0.56 (0.32–0.97) for DASH [29], which is similar to our findings in the MEC where the inverse association was stronger for squamous cell carcinoma. Previously, a European cohort study reported that an inverse association of fruit and vegetable consumption, essential components of the diet quality indexes, in current smokers was limited to squamous cell carcinomas [30,31]. A large case-control study in the US also found that the inverse association of the “fruits and vegetables” dietary pattern was more evident for squamous cell carcinoma [32]. Among the main histological types of non-small cell lung cancer, squamous cell carcinoma is most strongly associated with smoking [33]. Since most cases of squamous cell carcinoma (96%) were among ever smokers in the MEC, we could not examine the associations of diet quality with this cell type of lung cancer among never smokers.

The Mediterranean-style diet, which has been reported to be associated with a 16% (95% CI: 0.76–0.94) decreased risk of lung cancer in a recent meta-analysis [34], is known to have anti-inflammatory potential, lowering levels of inflammation in a randomized trial [35,36]. Indeed, a more pro-inflammatory diet assessed by the DII was associated with risk of lung cancer, but only among current smokers [37] or participants with a history of smoking [38,39] in prospective studies. However, in a cohort of heavy smokers enrolled in a lung cancer screening trial, the DII was not statistically significantly associated with risk of lung cancer [40]. In the MEC, although the DII was moderately to strongly correlated with HEI-2015 (r = −0.77), AHEI-2010 (−0.63), aMED (−0.48), and DASH (−0.64), the association for DII (10% decrease with the most anti-inflammatory diet) appeared to be weaker than for the other four indexes (15–17% decrease) overall. Unlike in the previous studies, we found no indication of heterogeneity in the associations across smoking status not only for the DII but also for the other indexes in the MEC.

The current study has many strengths, which include its prospective design, a large sample size with five racial/ethnic backgrounds, long years of follow-up, and a wide range of potential confounders available. Despite the comprehensive control for cigarette smoking and other potential confounders, residual confounding might still exist. However, diet quality was inversely associated with lung cancer risk among never smokers especially for the AHEI-2010, aMED, and DASH, indicating that our findings are not due to confounding by smoking. Measurement error in self-administered dietary assessment is inevitable. Dietary habits may change during the follow-up period. However, when analyzing data updated with a 10-year follow-up QFFQ, the associations did not substantially change.

## 5. Conclusions

In summary, our findings provide additional evidence that high-quality diets assessed by various dietary indexes are associated with reduced risk of incident lung cancer in both men and women. Our findings also suggest that the association may be stronger for squamous cell carcinoma than for other cell types but do not differ across sex, racial/ethnic groups and smoking status.

## Figures and Tables

**Table 1:** Pearson correlation coefficients between diet quality indexes in the Multiethnic Cohort Study, 1993–1996.

	AHEI-2010	aMED	DASH	DII
HEI-2015	0.63	0.53	0.72	−0.77
AHEI-2010	-	0.67	0.69	−0.63
aMED	-	-	0.62	−0.48
DASH	-	-	-	−0.64

AHEI-2010, Alternative Healthy Eating Index-2010; aMED, alternate Mediterranean Diet score; HEI-2015, Healthy Eating Index-2015; DASH, Dietary Approaches to Stop Hypertension score; DII, Dietary Inflammatory Index.

**Table 2:** Baseline characteristics of participants by quintile of Healthy Eating Index-2015 in the Multiethnic Cohort Study, 1993–1996.

	Healthy Eating Index-2015				
Characteristics	17.9−58.2	58.3−64.6	64.7−70.2	70.3−76.6	76.7−100
Men (*n* = 81,619)	(*n* = 20,323)	(*n* = 18,260)	(*n* = 16,329)	(*n* = 14,514)	(*n* = 12,193)
Age at cohort entry, years, mean (SD)	57.7 (8.7)	59.6 (8.8)	60.7 (8.8)	61.4 (8.7)	62.4 (8.5)
Race/ethnicity, *n* (%)					
African American	2136 (10.5)	2126 (11.6)	2155 (13.2)	2207 (15.2)	2167 (17.8)
Native Hawaiian	1861 (9.2)	1301 (7.1)	1006 (6.2)	857 (5.9)	695 (5.7)
Japanese American	7271 (35.8)	5843 (32.0)	4880 (29.9)	3872 (26.7)	3113 (25.5)
Latino	5064 (24.9)	4988 (27.3)	4104 (25.1)	3039 (20.9)	1820 (14.9)
White	3991 (19.6)	4002 (21.9)	4184 (25.6)	4539 (31.3)	4398 (36.1)
Family history of lung cancer, *n* (%)	1058 (5.2)	1024 (5.6)	964 (5.9)	854 (5.9)	732 (6.0)
Education, years, mean (SD)	12.9 (3.3)	13.0 (3.4)	13.3 (3.3)	13.7 (3.2)	14.1 (3.0)
Smoking status, *n* (%)					
Never	5107 (25.1)	5327 (29.2)	5219 (32.0)	4950 (34.1)	4620 (37.9)
Former	9247 (45.5)	9180 (50.3)	8616 (52.8)	7942 (54.7)	6644 (54.5)
Current	5969 (29.4)	3753 (20.6)	2494 (15.3)	1622 (11.2)	929 (7.6)
Pack-years among ever smokers, mean (SD)	23.6 (17.3)	20.9 (16.5)	19.7 (16.4)	18.9 (16.0)	17.9 (15.5)
Body mass index, kg/m^2^, mean (SD)	26.8 (4.3)	26.8 (4.1)	26.7 (4.0)	26.5 (3.9)	26.0 (3.7)
Obesity (BMI ≥ 30 kg/m^2^), *n* (%)	3965 (19.5)	3371 (18.5)	2786 (17.1)	2267 (15.6)	1539 (12.6)
Physical activity, h/day, mean (SD) ^1^	1.23 (1.52)	1.28 (1.49)	1.35 (1.51)	1.39 (1.46)	1.50 (1.51)
Total energy intake, kcal/day, mean (SD)	2495 (1170)	2517 (1191)	2461 (1137)	2355 (1043)	2183 (915)
Alcohol intake, g/day, mean (SD)	14.0 (35.8)	16.1 (35.7)	15.8 (33.3)	15.3 (29.6)	12.3 (23.0)
Women (*n* = 97,699)	(*n* = 15,327)	(*n* = 17,358)	(*n* = 19,461)	(*n* = 21,441)	(*n* = 24,112)
Age at cohort entry, years, mean (SD)	56.5 (8.5)	58.2 (8.7)	59.4 (8.7)	60.3 (8.7)	61.8 (8.5)
Race/ethnicity, *n* (%)					
African American	2204 (14.4)	2683 (15.5)	3466 (17.8)	4253 (19.8)	5919 (24.5)
Native Hawaiian	1534 (10.0)	1408 (8.1)	1386 (7.1)	1470 (6.9)	1498 (6.2)
Japanese American	4510 (29.4)	5324 (30.7)	5707 (29.3)	5899 (27.5)	6151 (25.5)
Latino	3981 (26.0)	4376 (25.2)	4310 (22.1)	3975 (18.5)	3193 (13.2)
White	3098 (20.2)	3567 (20.5)	4592 (23.6)	5844 (27.3)	7351 (30.5)
Family history of lung cancer, *n* (%)	982 (6.4)	1075 (6.2)	1319 (6.8)	1504 (7.0)	1795 (7.4)
Education, years, mean (SD)	12.5 (3.3)	12.7 (3.3)	13.0 (3.2)	13.4 (3.1)	13.8 (2.9)
Smoking status, *n* (%)					
Never	7636 (49.8)	9740 (56.1)	11,153 (57.3)	12,554 (58.6)	14,329 (59.4)
Former	3753 (24.5)	4602 (26.5)	5589 (28.7)	6472 (30.2)	7812 (32.4)
Current	3938 (25.7)	3016 (17.4)	2719 (14.0)	2415 (11.3)	1971 (8.2)
Pack-years among ever smokers, mean (SD)	18.5 (15.7)	16.1 (14.7)	15.4 (14.4)	14.3 (13.9)	13.7 (13.4)
Body mass index, kg/m^2^, mean (SD)	27.1 (5.9)	26.8 (5.6)	26.5 (5.5)	26.2 (5.3)	25.6 (5.1)
Obesity (BMI ≥ 30 kg/m^2^), *n* (%)	4110 (26.8)	4223 (24.3)	4390 (22.6)	4407 (20.6)	4086 (16.9)
Physical activity, h/day, mean (SD) ^1^	0.93 (1.21)	1.01 (1.24)	1.07 (1.23)	1.17 (1.27)	1.25 (1.28)
Total energy intake, kcal/day, mean (SD)	2053 (1063)	2041 (1025)	2008 (965)	1955 (914)	1860 (815)
Alcohol intake, g/day, mean (SD)	4.0 (17.3)	4.2 (16.1)	4.5 (15.9)	4.8 (14.6)	4.3 (11.8)

^1^ Moderate to vigorous activity.

**Table 3:** Diet quality indexes and lung cancer risk in the Multiethnic Cohort Study, 1993–2014.

	Men (*n* = 81,619)		Women (*n* = 97,699)		*P* for Heterogeneity	All (*n* = 179,318)	
	Cases	HR (95% CI) ^1^	Cases	HR (95% CI) ^1^		Cases	HR (95% CI) ^2^
HEI-2015							
17.9 to 58.2	926	1.00 (ref.)	481	1.00 (ref.)		1407	1.00 (ref.)
58.3 to 64.6	677	0.93 (0.84–1.03)	466	0.98 (0.86–1.12)		1143	0.95 (0.88–1.03)
64.7 to 70.2	531	0.89 (0.79–0.99)	504	0.97 (0.86–1.11)		1035	0.92 (0.85–1.00)
70.3 to 76.6	442	0.89 (0.79–1.00)	471	0.87 (0.76–0.99)		913	0.87 (0.80–0.95)
76.7 to 100	353	0.91 (0.80–1.04)	499	0.81 (0.71–0.92)		852	0.85 (0.77–0.93)
P for trend		0.046		<0.001	0.43		<0.001
AHEI-2010							
25.1 to 56.6	785	1.00 (ref.)	557	1.00 (ref.)		1342	1.00 (ref.)
56.7 to 62.2	627	0.98 (0.88–1.09)	496	0.95 (0.84–1.07)		1123	0.97 (0.89–1.05)
62.3 to 67.1	550	0.95 (0.85–1.06)	484	0.93 (0.82–1.06)		1034	0.94 (0.87–1.03)
67.2 to 72.6	501	0.95 (0.84–1.06)	469	0.90 (0.79–1.03)		970	0.93 (0.85–1.01)
72.7 to 104.5	466	0.87 (0.77–0.98)	415	0.81 (0.71–0.93)		881	0.84 (0.77–0.92)
P for trend		0.024		0.0022	0.70		<0.001
aMED							
0 to 2	722	1.00 (ref.)	640	1.00 (ref.)		1362	1.00 (ref.)
3	574	0.95 (0.85–1.06)	489	0.92 (0.82–1.04)		1063	0.94 (0.86–1.02)
4	567	0.89 (0.80–1.00)	421	0.82 (0.72–0.93)		988	0.86 (0.79–0.94)
5	441	0.80 (0.70–0.91)	405	0.86 (0.75–0.99)		846	0.83 (0.75–0.91)
6 to 9	625	0.87 (0.77–0.98)	466	0.78 (0.68–0.90)		1091	0.83 (0.76–0.91)
P for trend		0.0024		<0.001	0.95		<0.001
DASH							
8 to 20	850	1.00 (ref.)	678	1.00 (ref.)		1528	1.00 (ref.)
21 to 22	463	0.90 (0.81–1.01)	394	0.95 (0.84–1.08)		857	0.93 (0.85–1.01)
23 to 25	713	0.92 (0.83–1.02)	586	0.88 (0.78–0.98)		1299	0.90 (0.83–0.97)
26 to 27	368	0.84 (0.74–0.95)	363	0.93 (0.82–1.07)		731	0.89 (0.81–0.97)
28 to 40	535	0.92 (0.82–1.04)	400	0.73 (0.64–0.84)		935	0.83 (0.76–0.91)
P for trend		0.078		<0.001	0.23		<0.001
DII							
0.46 to 4.98	1055	1.00 (ref.)	476	1.00 (ref.)		1531	1.00 (ref.)
−0.94 to 0.45	656	0.92 (0.83–1.01)	463	0.96 (0.85–1.10)		1119	0.93 (0.86–1.01)
−2.12 to −0.95	479	0.88 (0.78–0.98)	491	0.97 (0.85–1.11)		970	0.92 (0.85–1.00)
−3.24 to −2.13	413	0.95 (0.85–1.07)	466	0.85 (0.74–0.97)		879	0.89 (0.81–0.97)
−6.44 to −3.25	326	0.91 (0.80–1.04)	525	0.90 (0.79–1.03)		851	0.90 (0.82–0.99)
P for trend		0.12		0.030	0.89		0.008

AHEI-2010, Alternative Healthy Eating Index-2010; aMED, alternate Mediterranean Diet score, DASH, Dietary Approaches to Stop Hypertension score; DII, Dietary Inflammatory Index. ^1^ Adjusted by Cox regression with as the time metric for age at cohort entry, race/ethnicity, family history of lung cancer, education, BMI, physical activity, and total energy intake in the smoking model, which included smoking status, average number of cigarettes per day, squared average number of cigarettes per day, number of years smoked (time-dependent), number of years since quitting (time-dependent), and interactions between race/ethnicity and smoking status, average number of cigarettes per day, squared average number of cigarettes per day and number of years smoked. For HEI-2015 and DAHS, additionally adjusted for alcohol intake. ^2^ Further adjusted for sex as baseline hazard strata variable.

**Table 4:** Diet quality indexes and lung cancer risk by histologic type in the Multiethnic Cohort Study, 1993–2014.

	Adenocarcinoma		Squamous Cell		Small Cell		Large Cell		Other/Unspecified		*P* for Heterogeneity
	Cases	HR (95% CI) ^1^	Cases	HR (95% CI) ^1^	Cases	HR (95% CI) ^1^	Cases	HR (95% CI) ^1^	Cases	HR (95% CI) ^1^	
HEI-2015											
17.9 to 58.2	506	1.00 (ref.)	326	1.00 (ref.)	167	1.00 (ref.)	44	1.00 (ref.)	364	1.00 (ref.)	
58.3 to 64.6	424	0.92 (0.81–1.05)	237	0.91 (0.76–1.07)	119	0.95 (0.75–1.21)	34	0.94 (0.60–1.48)	329	1.03 (0.88–1.20)	
64.7 to 70.2	417	0.93 (0.81–1.06)	212	0.93 (0.78–1.12)	94	0.89 (0.69–1.16)	33	1.01 (0.64–1.61)	279	0.92 (0.78–1.08)	
70.3 to 76.6	418	0.94 (0.82–1.08)	152	0.78 (0.63–0.95)	77	0.87 (0.66–1.16)	24	0.82 (0.49–1.39)	242	0.85 (0.72–1.01)	
76.7 to 100	393	0.88 (0.76–1.02)	131	0.75 (0.60–0.93)	89	1.20 (0.91–1.59)	24	0.88 (0.51–1.51)	215	0.76 (0.63–0.91)	
P for trend		0.14		0.004		0.62		0.54		<0.001	<0.001
AHEI-2010											
25.1 to 56.6	474	1.00 (ref.)	294	1.00 (ref.)	168	1.00 (ref.)	46	1.00 (ref.)	360	1.00 (ref.)	
56.7 to 62.2	432	1.00 (0.88–1.14)	238	1.00 (0.84–1.19)	114	0.88 (0.69–1.12)	34	0.85 (0.55–1.34)	305	0.96 (0.82–1.11)	
62.3 to 67.1	430	1.02 (0.89–1.17)	208	0.97 (0.81–1.16)	87	0.75 (0.57–0.97)	23	0.65 (0.39–1.08)	286	0.95 (0.81–1.11)	
67.2 to 72.6	407	0.98 (0.86–1.13)	182	0.92 (0.76–1.11)	94	0.91 (0.70–1.18)	26	0.80 (0.48–1.31)	261	0.90 (0.76–1.06)	
72.7 to 104.5	415	0.96 (0.84–1.11)	136	0.71 (0.58–0.88)	83	0.88 (0.66–1.16)	30	0.96 (0.59–1.57)	217	0.75 (0.63–0.89)	
P for trend		0.61		0.003		0.27		0.65		0.002	<0.001
aMED											
0 to 2	501	1.00 (ref.)	272	1.00 (ref.)	162	1.00 (ref.)	45	1.00 (ref.)	382	1.00 (ref.)	
3	415	0.98 (0.86–1.12)	225	1.00 (0.83–1.19)	104	0.81 (0.63–1.04)	31	0.83 (0.52–1.31)	288	0.90 (0.77–1.05)	
4	399	0.92 (0.80–1.05)	204	0.90 (0.75–1.09)	98	0.79 (0.61–1.03)	26	0.70 (0.42–1.15)	261	0.80 (0.68–0.94)	
5	350	0.90 (0.78–1.04)	164	0.82 (0.67–1.01)	83	0.75 (0.56–1.00)	26	0.84 (0.50–1.42)	223	0.76 (0.64–0.91)	
6 to 9	493	0.97 (0.84–1.12)	193	0.77 (0.62–0.95)	99	0.74 (0.56–0.99)	31	0.79 (0.47–1.36)	275	0.73 (0.61–0.87)	
P for trend		0.43		0.004		0.037		0.42		<0.001	0.0038
DASH											
8 to 20	552	1.00 (ref.)	347	1.00 (ref.)	182	1.00 (ref.)	46	1.00 (ref.)	401	1.00 (ref.)	
21 to 22	328	0.94 (0.82–1.08)	169	0.85 (0.70–1.02)	78	0.82 (0.63–1.07)	26	0.97 (0.60–1.59)	256	1.01 (0.86–1.18)	
23 to 25	535	0.96 (0.84–1.08)	246	0.83 (0.70–0.98)	146	1.07 (0.85–1.34)	35	0.82 (0.52–1.30)	337	0.83 (0.72–0.97)	
26 to 27	309	0.93 (0.81–1.08)	136	0.83 (0.67–1.02)	62	0.87 (0.64–1.18)	28	1.16 (0.70–1.92)	196	0.83 (0.70–1.00)	
28 to 40	434	0.93 (0.81–1.07)	160	0.77 (0.63–0.95)	78	0.91 (0.68–1.22)	24	0.77 (0.45–1.32)	239	0.73 (0.61–0.87)	
P for trend		0.31		0.006		0.61		0.44		<0.001	<0.001
DII											
0.46 to 4.98	536	1.00 (ref.)	362	1.00 (ref.)	187	1.00 (ref.)	44	1.00 (ref.)	402	1.00 (ref.)	
−0.94 to 0.45	422	0.93 (0.82–1.06)	235	0.91 (0.77–1.08)	107	0.84 (0.66–1.07)	48	1.42 (0.94–2.16)	307	0.94 (0.81–1.09)	
−2.12 to −0.95	405	0.97 (0.85–1.11)	191	0.92 (0.76–1.10)	87	0.85 (0.65–1.10)	15	0.53 (0.29–0.97)	272	0.93 (0.79–1.09)	
−3.24 to −2.13	391	0.95 (0.82–1.09)	138	0.76 (0.62–0.94)	80	0.92 (0.70–1.22)	26	1.07 (0.64–1.78)	244	0.88 (0.74–1.04)	
−6.44 to −3.25	404	0.98 (0.86–1.13)	132	0.81 (0.66–1.01)	85	1.15 (0.87–1.52)	26	1.19 (0.71–2.02)	204	0.75 (0.63–0.91)	
P for trend		0.83		0.01		0.68		0.93		0.004	<0.001

^1^ Adjusted by Cox regression with age as the time metric for age at cohort entry, sex, race/ethnicity, family history of lung cancer, education, BMI, physical activity, and total energy intake in the smoking model. For HEI-2015 and DAHS, additionally adjusted for alcohol intake.

## Data Availability

The data presented in this study are available on request from the Multiethnic Cohort (MEC) Study. The data are not publicly available because they contain protected health information. All data requests will be reviewed by the MEC Study Research Committee (see https://www.uhcancercenter.org/for-researchers/mec-data-sharing, accessed on 1 April 2021).

## Supplementary Materials

The following are available online at https://www.mdpi.com/article/10.3390/nu13051614/s1, Table S1: Baseline characteristics of participants by lowest (Q1) and highest (Q5) quintiles of the diet quality indexes in the Multiethnic Cohort Study, 1993–1996, Table S2: Diet quality indexes and lung cancer risk by race/ethnicity in the Multiethnic Cohort Study, 1993-2014, Table S3: Diet quality indexes and lung cancer risk by smoking status in the Multiethnic Cohort Study, 1993–2014, Table S4: Diet quality indexes as time-dependent variables and lung cancer risk in the Multiethnic Cohort Study, 1993–2014.
