# Supplementary material for: Diet Quality and Risk of Lung Cancer in the Multiethnic Cohort Study
Source: Nutrients. 2021 May 12;13(5):1614. doi: 10.3390/nu13051614 (PMC8151689; doi:10.3390/nu13051614)
Supplement: Supplementary file 1 [file nutrients-13-01614-s001.zip › nutrients-1188204-supplementary.pdf]

**Supplementary Tables**

**Supplementary Table S1.** Baseline characteristics of participants by lowest (Q1) and highest (Q5) quintiles of the diet quality indexes in the Multiethnic Cohort Study, 1993-1996

| | AHEI-2010 | | aMED | | DASH | | DII | |
|---|---|---|---|---|---|---|---|---|
| | Q1 (25.1 to 56.6) | Q5 (72.7 to 104.5) | Q1 (0 to 2) | Q5 (6 to 9) | Q1 (8 to 20) | Q5 (28 to 40) | Q5 (0.46 to 4.98) | Q1 (-6.44 to -3.25) |
| **Men, n** | | | | | | | | |
| Age at cohort entry, y, mean (SD) | 58.0 (8.8) | 61.6 (8.8) | 59.2 (8.8) | 60.7 (8.9) | 57.3 (8.6) | 62.1 (8.6) | 57.6 (8.7) | 63.0 (8.5) |
| Race/ethnicity, n (%) | | | | | | | | |
| African American | 2584 (14.4) | 1702 (10.7) | 2464 (15.4) | 2509 (12.2) | 2511 (14.0) | 2177 (11.5) | 2998 (13.4) | 1505 (13.2) |
| Native Hawaiian | 1310 (7.3) | 1047 (6.6) | 1032 (6.5) | 1622 (7.9) | 1833 (10.2) | 931 (4.9) | 2048 (9.1) | 696 (6.1) |
| Japanese American | 4576 (25.5) | 6160 (38.6) | 4329 (27.1) | 6936 (33.7) | 7503 (41.8) | 4485 (23.7) | 7278 (32.4) | 3767 (33.1) |
| Latino | 4945 (27.5) | 2246 (14.1) | 4171 (26.1) | 3816 (18.5) | 3230 (18.0) | 4419 (23.4) | 4936 (22.0) | 1784 (15.7) |
| White | 4560 (25.4) | 4807 (30.1) | 3961 (24.8) | 5714 (27.7) | 2867 (16.0) | 6911 (36.5) | 5188 (23.1) | 3625 (31.9) |
| Family history of lung cancer, n (%) | 978 (5.4) | 1019 (6.4) | 887 (5.6) | 1231 (6.0) | 955 (5.3) | 1100 (5.8) | 1265 (5.6) | 647 (5.7) |
| Education, y, mean (SD) | 13.0 (3.3) | 14.0 (3.0) | 13.1 (3.3) | 13.7 (3.2) | 13.1 (3.1) | 13.8 (3.4) | 13.1 (3.1) | 13.9 (3.1) |
| Smoking status, n (%) | | | | | | | | |
| Never | 4753 (26.4) | 5379 (33.7) | 4403 (27.6) | 7007 (34.0) | 4329 (24.1) | 7078 (37.4) | 5426 (24.2) | 4227 (37.2) |
| Former | 8095 (45.0) | 9021 (56.5) | 7456 (46.7) | 11081 (53.8) | 8216 (45.8) | 10246 (54.1) | 10295 (45.9) | 6346 (55.8) |
| Current | 5127 (28.5) | 1562 (9.8) | 4098 (25.7) | 2509 (12.2) | 5399 (30.1) | 1599 (8.5) | 6727 (30.0) | 804 (7.1) |
| Pack-years among ever smokers, mean (SD) | 23.2 (17.3) | 19.0 (15.8) | 22.6 (17.2) | 18.8 (15.9) | 23.5 (16.8) | 18.0 (15.9) | 24.0 (17.3) | 18.1 (15.6) |
| Body mass index, kg/m$^2$, mean (SD) | 26.8 (4.2) | 26.0 (3.8) | 26.8 (4.1) | 26.4 (4.0) | 26.8 (4.2) | 26.1 (3.8) | 26.8 (4.3) | 26.0 (3.8) |
| Obesity (BMI ≥30 kg/m$^2$), n (%) | 3361 (18.7) | 2007 (12.6) | 2944 (18.4) | 3115 (15.1) | 3333 (18.6) | 2539 (13.4) | 4337 (19.3) | 1447 (12.7) |
| Physical activity, h/d, mean (SD)* | 1.22 (1.52) | 1.50 (1.48) | 1.09 (1.37) | 1.58 (1.60) | 1.23 (1.50) | 1.49 (1.53) | 1.29 (1.55) | 1.45 (1.50) |
| Total energy intake, kcal/d, mean (SD) | 2331 (1109) | 2475 (997) | 1790 (766) | 3049 (1222) | 2182 (921) | 2651 (1191) | 2571 (1185) | 2222 (954) |
| Alcohol intake, g/d, mean (SD) | 24.8 (52.8) | 10.9 (14.2) | 16.0 (39.6) | 13.5 (25.5) | 18.6 (39.9) | 11.6 (24.6) | 25.2 (51.2) | 7.5 (14.0) |
| | | | | | | | | |
| **Women, n** | | | | | | | | |
| Age at cohort entry, y, mean (SD) | 57.2 (8.7) | 61.1 (8.7) | 58.4 (8.9) | 60.5 (8.7) | 56.8 (8.6) | 61.6 (8.5) | 56.0 (8.5) | 61.9 (8.5) |
| Race/ethnicity, n (%) | | | | | | | | |
| African American | 3657 (20.7) | 3517 (17.2) | 4008 (19.5) | 4488 (19.2) | 4668 (22.1) | 3645 (16.3) | 2786 (20.3) | 4622 (18.8) |
| Native Hawaiian | 1423 (8.0) | 1480 (7.2) | 1342 (6.5) | 2109 (9.0) | 2232 (10.5) | 1297 (5.8) | 1481 (10.8) | 1676 (6.8) |
| Japanese American | 3826 (21.6) | 7523 (36.8) | 5007 (24.4) | 7359 (31.4) | 7184 (33.9) | 5264 (23.6) | 3392 (24.7) | 7959 (32.4) |
| Latino | 4382 (24.8) | 2271 (11.1) | 4687 (22.8) | 3780 (16.1) | 3754 (17.7) | 4619 (20.7) | 2766 (20.2) | 3637 (14.8) |

| | AHEI-2010 | | aMED | | DASH | | DII | |
|---|---|---|---|---|---|---|---|---|
| | Q1 (25.1 to 56.6) | Q5 (72.7 to 104.5) | Q1 (0 to 2) | Q5 (6 to 9) | Q1 (8 to 20) | Q5 (28 to 40) | Q5 (0.46 to 4.98) | Q1 (-6.44 to -3.25) |
| White | 4409 (24.9) | 5665 (27.7) | 5504 (26.8) | 5684 (24.3) | 3331 (15.7) | 7472 (33.5) | 3291 (24.0) | 6668 (27.1) |
| Family history of lung cancer, n (%) | 1158 (6.5) | 1534 (7.5) | 1335 (6.5) | 1673 (7.1) | 1383 (6.5) | 1622 (7.3) | 921 (6.7) | 1765 (7.2) |
| Education, y, mean (SD) | 12.8 (3.2) | 13.7 (2.9) | 12.9 (3.1) | 13.4 (3.1) | 12.9 (3.0) | 13.5 (3.2) | 13.0 (2.9) | 13.5 (3.1) |
| Smoking status, n (%) | | | | | | | | |
| Never | 9136 (51.6) | 11800 (57.7) | 10864 (52.9) | 13993 (59.7) | 10669 (50.4) | 13534 (60.7) | 6325 (46.1) | 15098 (61.5) |
| Former | 4490 (25.4) | 6781 (33.1) | 5581 (27.2) | 7131 (30.4) | 5300 (25.0) | 7084 (31.8) | 3472 (25.3) | 7452 (30.3) |
| Current | 4071 (23.0) | 1875 (9.2) | 4103 (20.0) | 2296 (9.8) | 5200 (24.6) | 1679 (7.5) | 3919 (28.6) | 2012 (8.2) |
| Pack-years among ever smokers, mean (SD) | 18.0 (15.6) | 14.2 (13.6) | 17.2 (15.3) | 13.5 (13.3) | 17.4 (14.8) | 13.5 (13.6) | 19.2 (15.8) | 13.5 (13.3) |
| Body mass index, kg/m$^2$, mean (SD) | 27.2 (5.8) | 25.4 (5.2) | 26.7 (5.6) | 26.0 (5.4) | 27.0 (5.8) | 25.5 (5.1) | 27.2 (6.1) | 25.6 (5.1) |
| Obesity (BMI ≥30 kg/m$^2$), n (%) | 4741 (26.8) | 3397 (16.6) | 4896 (23.8) | 4526 (19.3) | 5502 (26.0) | 3812 (17.1) | 3792 (27.6) | 4182 (17.0) |
| Physical activity, h/d, mean (SD)* | 0.97 (1.22) | 1.27 (1.30) | 0.94 (1.15) | 1.28 (1.35) | 0.93 (1.17) | 1.29 (1.34) | 0.98 (1.22) | 1.22 (1.28) |
| Total energy intake, kcal/d, mean (SD) | 1760 (885) | 2120 (867) | 1391 (577) | 2587 (1074) | 1681 (730) | 2269 (1041) | 2116 (1058) | 1890 (856) |
| Alcohol intake, g/d, mean (SD) | 6.6 (24.7) | 4.3 (8.8) | 4.6 (17.5) | 4.2 (12.5) | 4.8 (18.5) | 4.0 (12.3) | 9.1 (29.9) | 2.9 (8.0) |

*Moderate to vigorous activity

**Supplementary Table S2.** Diet quality indexes and lung cancer risk by race/ethnicity in the Multiethnic Cohort Study, 1993-2014

| | African American (n=29,316) | | Native Hawaiian (n=13,016) | | Japanese American (n=52,570) | | Latino (n=38,850) | | White (n=45,566) | | P for heterogeneity |
|---|---|---|---|---|---|---|---|---|---|---|---|
| | Cases | HR (95% CI)* | Cases | HR (95% CI)* | Cases | HR (95% CI)* | Cases | HR (95% CI)* | Cases | HR (95% CI)* | |
| HEI-2015 | | | | | | | | | | | |
| 17.9 to 58.2 | 237 | 1.00 (ref.) | 193 | 1.00 (ref.) | 413 | 1.00 (ref.) | 224 | 1.00 (ref.) | 340 | 1.00 (ref.) | |
| 58.3 to 64.6 | 265 | 1.12 (0.94-1.34) | 100 | 0.78 (0.61-1.00) | 327 | 0.99 (0.86-1.15) | 170 | 0.87 (0.71-1.07) | 281 | 0.91 (0.77-1.06) | |
| 64.7 to 70.2 | 254 | 1.04 (0.86-1.24) | 99 | 1.00 (0.77-1.28) | 264 | 0.95 (0.81-1.11) | 148 | 0.93 (0.75-1.15) | 270 | 0.82 (0.69-0.96) | |
| 70.3 to 76.6 | 222 | 0.91 (0.75-1.10) | 67 | 0.74 (0.55-0.99) | 203 | 0.89 (0.74-1.06) | 104 | 0.81 (0.64-1.03) | 317 | 0.92 (0.78-1.07) | |
| 76.7 to 100 | 253 | 0.91 (0.75-1.10) | 58 | 0.84 (0.61-1.16) | 184 | 0.91 (0.75-1.09) | 68 | 0.77 (0.58-1.02) | 289 | 0.80 (0.68-0.95) | |
| P for trend | | 0.08 | | 0.15 | | 0.16 | | 0.048 | | 0.02 | 0.96 |
| AHEI-2010 | | | | | | | | | | | |
| 25.1 to 56.6 | 299 | 1.00 (ref.) | 138 | 1.00 (ref.) | 277 | 1.00 (ref.) | 231 | 1.00 (ref.) | 397 | 1.00 (ref.) | |
| 56.7 to 62.2 | 270 | 1.05 (0.89-1.24) | 100 | 0.88 (0.68-1.14) | 277 | 1.04 (0.88-1.23) | 162 | 0.78 (0.64-0.96) | 314 | 1.00 (0.86-1.16) | |
| 62.3 to 67.1 | 256 | 1.09 (0.92-1.29) | 94 | 0.88 (0.67-1.16) | 249 | 0.85 (0.72-1.02) | 143 | 0.87 (0.70-1.07) | 292 | 0.97 (0.83-1.13) | |
| 67.2 to 72.6 | 225 | 1.00 (0.84-1.20) | 111 | 1.11 (0.86-1.45) | 293 | 0.91 (0.77-1.08) | 109 | 0.86 (0.68-1.08) | 232 | 0.85 (0.72-1.00) | |
| 72.7 to 104.5 | 181 | 0.92 (0.76-1.11) | 74 | 0.81 (0.60-1.09) | 295 | 0.79 (0.66-0.94) | 69 | 0.81 (0.61-1.06) | 262 | 0.88 (0.75-1.04) | |
| P for trend | | 0.49 | | 0.53 | | 0.003 | | 0.11 | | 0.04 | 0.29 |
| aMED | | | | | | | | | | | |
| 0 to 2 | 307 | 1.00 (ref.) | 122 | 1.00 (ref.) | 285 | 1.00 (ref.) | 210 | 1.00 (ref.) | 438 | 1.00 (ref.) | |
| 3 | 244 | 0.96 (0.81-1.14) | 95 | 0.96 (0.73-1.26) | 286 | 1.07 (0.91-1.26) | 151 | 0.87 (0.70-1.08) | 287 | 0.86 (0.74-1.00) | |
| 4 | 226 | 0.90 (0.75-1.08) | 92 | 0.81 (0.61-1.08) | 258 | 0.86 (0.72-1.03) | 126 | 0.76 (0.61-0.96) | 286 | 0.90 (0.78-1.06) | |
| 5 | 207 | 0.93 (0.77-1.13) | 76 | 0.70 (0.51-0.95) | 228 | 0.88 (0.73-1.06) | 110 | 0.78 (0.61-1.01) | 225 | 0.79 (0.67-0.94) | |
| 6 to 9 | 247 | 0.82 (0.67-1.00) | 132 | 0.86 (0.64-1.16) | 334 | 0.91 (0.76-1.10) | 117 | 0.84 (0.65-1.09) | 261 | 0.76 (0.64-0.91) | |
| P for trend | | 0.07 | | 0.13 | | 0.10 | | 0.09 | | 0.002 | 0.60 |
| DASH | | | | | | | | | | | |
| 8 to 20 | 378 | 1.00 (ref.) | 205 | 1.00 (ref.) | 482 | 1.00 (ref.) | 166 | 1.00 (ref.) | 297 | 1.00 (ref.) | |
| 21 to 22 | 216 | 0.97 (0.82-1.15) | 84 | 1.01 (0.78-1.32) | 212 | 0.86 (0.73-1.02) | 123 | 0.94 (0.75-1.19) | 222 | 0.90 (0.75-1.07) | |
| 23 to 25 | 290 | 0.88 (0.75-1.03) | 108 | 0.89 (0.70-1.14) | 315 | 0.91 (0.79-1.06) | 183 | 0.83 (0.67-1.03) | 403 | 0.92 (0.79-1.07) | |
| 26 to 27 | 162 | 0.90 (0.74-1.09) | 54 | 0.93 (0.68-1.29) | 165 | 0.87 (0.72-1.05) | 116 | 0.89 (0.69-1.14) | 234 | 0.85 (0.71-1.02) | |
| 28 to 40 | 185 | 0.79 (0.65-0.96) | 66 | 0.95 (0.70-1.30) | 217 | 0.93 (0.78-1.11) | 126 | 0.75 (0.58-0.96) | 341 | 0.79 (0.66-0.93) | |
| P for trend | | 0.02 | | 0.57 | | 0.24 | | 0.03 | | 0.006 | 0.72 |
| DII | | | | | | | | | | | |
| 0.46 to 4.98 | 326 | 1.00 (ref.) | 183 | 1.00 (ref.) | 389 | 1.00 (ref.) | 212 | 1.00 (ref.) | 421 | 1.00 (ref.) | |
| -0.94 to 0.45 | 278 | 1.07 (0.91-1.26) | 107 | 1.00 (0.78-1.27) | 290 | 0.95 (0.81-1.11) | 162 | 0.78 (0.63-0.96) | 282 | 0.88 (0.76-1.03) | |
| -2.12 to -0.95 | 232 | 0.97 (0.82-1.16) | 85 | 1.06 (0.81-1.38) | 234 | 0.86 (0.72-1.01) | 154 | 0.92 (0.74-1.14) | 265 | 0.90 (0.77-1.05) | |
| -3.24 to -2.13 | 202 | 0.93 (0.78-1.12) | 73 | 1.01 (0.75-1.34) | 231 | 0.94 (0.79-1.12) | 103 | 0.78 (0.61-0.99) | 270 | 0.84 (0.71-0.98) | |
| -6.44 to -3.25 | 193 | 0.92 (0.76-1.12) | 69 | 1.01 (0.75-1.37) | 247 | 0.94 (0.79-1.12) | 83 | 0.92 (0.70-1.20) | 259 | 0.82 (0.70-0.97) | |
| P for trend | | 0.25 | | 0.87 | | 0.40 | | 0.28 | | 0.02 | 0.81 |

*Adjusted by Cox regression with age as the time metric for age at cohort entry, sex, family history of lung cancer, education, BMI, physical activity, and total energy intake in the smoking model. For HEI-2015 and DAHS, additionally adjusted for alcohol intake.

**Supplementary Table S3.** Diet quality indexes and lung cancer risk by smoking status in the Multiethnic Cohort Study, 1993-2014

| | Never smokers (n=80,635) | | Former smokers (n=69,857) | | Current smokers (n=28,826) | | P for heterogeneity |
|---|---|---|---|---|---|---|---|
| | Cases | HR (95% CI)* | Cases | HR (95% CI)* | Cases | HR (95% CI)* | |
| HEI-2015 | | | | | | | |
| 17.9 to 58.2 | 85 | 1.00 (ref.) | 430 | 1.00 (ref.) | 892 | 1.00 (ref.) | |
| 58.3 to 64.6 | 125 | 1.12 (0.85-1.48) | 442 | 0.95 (0.83-1.09) | 576 | 0.94 (0.85-1.05) | |
| 64.7 to 70.2 | 135 | 1.04 (0.79-1.37) | 443 | 0.90 (0.78-1.03) | 457 | 0.95 (0.84-1.06) | |
| 70.3 to 76.6 | 123 | 0.82 (0.62-1.09) | 457 | 0.90 (0.78-1.03) | 333 | 0.91 (0.80-1.03) | |
| 76.7 to 100 | 167 | 0.94 (0.72-1.24) | 438 | 0.83 (0.72-0.95) | 247 | 0.88 (0.76-1.02) | |
| P for trend | | 0.18 | | 0.0065 | | 0.052 | 0.92 |
| AHEI-2010 | | | | | | | |
| 25.1 to 56.6 | 110 | 1.00 (ref.) | 401 | 1.00 (ref.) | 831 | 1.00 (ref.) | |
| 56.7 to 62.2 | 117 | 0.83 (0.64-1.08) | 435 | 1.03 (0.90-1.18) | 571 | 0.96 (0.86-1.07) | |
| 62.3 to 67.1 | 130 | 0.82 (0.63-1.06) | 443 | 1.01 (0.88-1.15) | 461 | 0.95 (0.85-1.07) | |
| 67.2 to 72.6 | 156 | 0.91 (0.71-1.18) | 440 | 0.97 (0.84-1.11) | 374 | 0.91 (0.81-1.04) | |
| 72.7 to 104.5 | 122 | 0.66 (0.50-0.87) | 491 | 0.93 (0.81-1.07) | 268 | 0.85 (0.74-0.98) | |
| P for trend | | 0.015 | | 0.20 | | 0.020 | 0.19 |
| aMED | | | | | | | |
| 0 to 2 | 120 | 1.00 (ref.) | 452 | 1.00 (ref.) | 790 | 1.00 (ref.) | |
| 3 | 122 | 1.04 (0.80-1.34) | 429 | 1.01 (0.89-1.16) | 512 | 0.87 (0.78-0.97) | |
| 4 | 121 | 0.89 (0.69-1.16) | 418 | 0.89 (0.77-1.02) | 449 | 0.85 (0.76-0.96) | |
| 5 | 113 | 0.83 (0.63-1.09) | 372 | 0.85 (0.73-0.98) | 361 | 0.84 (0.74-0.97) | |
| 6 to 9 | 159 | 0.79 (0.60-1.04) | 539 | 0.89 (0.77-1.02) | 393 | 0.83 (0.72-0.95) | |
| P for trend | | 0.034 | | 0.020 | | 0.0048 | 0.74 |
| DASH | | | | | | | |
| 8 to 20 | 105 | 1.00 (ref.) | 461 | 1.00 (ref.) | 962 | 1.00 (ref.) | |
| 21 to 22 | 106 | 1.23 (0.94-1.62) | 315 | 0.88 (0.76-1.02) | 436 | 0.92 (0.82-1.03) | |
| 23 to 25 | 161 | 0.96 (0.75-1.24) | 575 | 0.89 (0.78-1.01) | 563 | 0.92 (0.82-1.02) | |
| 26 to 27 | 107 | 0.94 (0.71-1.24) | 350 | 0.85 (0.74-0.99) | 274 | 0.93 (0.81-1.07) | |
| 28 to 40 | 156 | 0.83 (0.64-1.09) | 509 | 0.83 (0.72-0.95) | 270 | 0.88 (0.76-1.02) | |
| P for trend | | 0.049 | | 0.0077 | | 0.059 | 0.83 |
| DII | | | | | | | |
| 0.46 to 4.98 | 75 | 1.00 (ref.) | 469 | 1.00 (ref.) | 987 | 1.00 (ref.) | |
| -0.94 to 0.45 | 109 | 1.03 (0.77-1.39) | 438 | 0.93 (0.82-1.07) | 572 | 0.95 (0.85-1.05) | |
| -2.12 to -0.95 | 140 | 1.04 (0.78-1.39) | 436 | 0.95 (0.83-1.08) | 394 | 0.90 (0.80-1.01) | |
| -3.24 to -2.13 | 148 | 0.96 (0.72-1.28) | 429 | 0.89 (0.78-1.02) | 302 | 0.91 (0.79-1.04) | |
| -6.44 to -3.25 | 163 | 0.90 (0.68-1.20) | 438 | 0.91 (0.80-1.05) | 250 | 0.95 (0.82-1.10) | |
| P for trend | | 0.32 | | 0.15 | | 0.14 | 0.48 |

*Adjusted by Cox regression with age as the time metric for age at cohort entry, sex, race/ethnicity, family history of lung cancer, education, BMI, physical activity, and total energy intake in the smoking model. For HEI-2015 and DAHS, additionally adjusted for alcohol intake.

**Supplementary Table S4.** Diet quality indexes as time-dependent variables* and lung cancer risk in the Multiethnic Cohort Study, 1993-2014

| | All participants (n=179,318) | | Respondents to the 10-year survey (n=82,119)† | |
|---|---|---|---|---|
| | Cases | HR (95% CI)‡ | Cases | HR (95% CI)‡ |
| HEI-2015 | | | | |
| 17.9 to 58.2 | 1306 | 1.00 (ref.) | 256 | 1.00 (ref.) |
| 58.3 to 64.6 | 1134 | 0.98 (0.91-1.06) | 258 | 0.97 (0.82-1.16) |
| 64.7 to 70.2 | 1031 | 0.94 (0.86-1.02) | 250 | 0.91 (0.76-1.09) |
| 70.3 to 76.6 | 951 | 0.90 (0.83-0.98) | 281 | 0.94 (0.79-1.12) |
| 76.7 to 100 | 928 | 0.86 (0.78-0.94) | 308 | 0.88 (0.74-1.05) |
| P for trend | | <0.001 | | 0.20 |
| AHEI-2010 | | | | |
| 25.1 to 56.6 | 1298 | 1.00 (ref.) | 286 | 1.00 (ref.) |
| 56.7 to 62.2 | 1091 | 0.95 (0.88-1.03) | 238 | 0.84 (0.71-1.01) |
| 62.3 to 67.1 | 999 | 0.91 (0.84-0.99) | 221 | 0.75 (0.63-0.90) |
| 67.2 to 72.6 | 1004 | 0.92 (0.85-1.00) | 282 | 0.85 (0.72-1.01) |
| 72.7 to 104.5 | 958 | 0.81 (0.74-0.88) | 326 | 0.73 (0.62-0.87) |
| P for trend | | <0.001 | | 0.0022 |
| aMED | | | | |
| 0 to 2 | 1367 | 1.00 (ref.) | 319 | 1.00 (ref.) |
| 3 | 1051 | 0.93 (0.86-1.01) | 263 | 0.92 (0.78-1.09) |
| 4 | 1012 | 0.88 (0.81-0.96) | 273 | 0.92 (0.78-1.08) |
| 5 | 860 | 0.84 (0.77-0.92) | 229 | 0.85 (0.71-1.01) |
| 6 to 9 | 1060 | 0.80 (0.73-0.88) | 269 | 0.73 (0.61-0.87) |
| P for trend | | <0.001 | | <0.001 |
| DASH | | | | |
| 8 to 20 | 1399 | 1.00 (ref.) | 277 | 1.00 (ref.) |
| 21 to 22 | 871 | 0.98 (0.90-1.06) | 206 | 0.95 (0.79-1.15) |
| 23 to 25 | 1352 | 0.94 (0.87-1.02) | 357 | 0.95 (0.81-1.11) |
| 26 to 27 | 757 | 0.90 (0.82-0.99) | 216 | 0.90 (0.75-1.08) |
| 28 to 40 | 971 | 0.80 (0.73-0.88) | 297 | 0.75 (0.63-0.90) |
| P for trend | | <0.001 | | 0.0024 |
| DII | | | | |
| 0.46 to 4.98 | 1405 | 1.00 (ref.) | 249 | 1.00 (ref.) |
| -0.94 to 0.45 | 1086 | 0.92 (0.85-1.00) | 229 | 0.84 (0.70-1.01) |
| -2.12 to -0.95 | 980 | 0.91 (0.84-0.99) | 274 | 0.97 (0.81-1.16) |
| -3.24 to -2.13 | 904 | 0.87 (0.80-0.95) | 246 | 0.83 (0.69-1.00) |
| -6.44 to -3.25 | 975 | 0.89 (0.82-0.98) | 355 | 0.93 (0.78-1.10) |
| P for trend | | 0.0032 | | 0.65 |

*Diet quality indexes were updated as time-dependent variables using data from the 10-year follow-up questionnaire for 82,119 participants who had no lung cancer prior to the 10-year follow-up survey.
†The outcome was lung cancer incidence occurred after the 10-year follow-up survey.
‡Adjusted by Cox regression with age as the time metric for age at cohort entry, sex, race/ethnicity, family history of lung cancer, education, BMI, physical activity, and total energy intake in the smoking model. For HEI-2015 and DAHS, additionally adjusted for alcohol intake.
